# Development of a Methylene Blue Carrier System in Gellan Gum for Application in Antimicrobial Photodynamic Therapy against *Candida albicans*

**DOI:** 10.1021/acsomega.5c02563

**Published:** 2025-10-15

**Authors:** Gabriela Vieira Mendes, Lívia Mara Alves Figueiredo Godoi, Éric Yukio Morissugui, Evelyn Luzia de Souza Santos, Amanda Siqueira Fraga, Dayane Batista Tada, Aguinaldo S. Garcez, Maíra Terra Garcia, Ganeshkumar Arumugam, Juliana Campos Junqueira

**Affiliations:** † Department of Biosciences and Oral Diagnosis, Institute of Science and Technology, São Paulo State University (Unesp), Av. Engenheiro Francisco José Longo, 777—Jardim São Dimas, São José dos Campos, São Paulo 12245-000, Brazil; ‡ São Carlos Institute of Physics, University of São Paulo, IFSC-USP, Sao Carlos, São Paulo 13566-590, Brazil; § Laboratory of Nanomaterials and Nanotoxicology, Universidade Federal de São Paulo (UNIFESP), R. Talim, 330—Vila Nair, São José dos Campos, São Paulo 12231-280, Brazil; ∥ Department of Oral Microbiology, 130267 Faculdade São Leopoldo Mandic, R. Dr. José Rocha Junqueira, 13, Ponte. Preta, Campinas, São Paulo 13045-755, Brazil; ⊥ Department of Materials Physics, Saveetha School of Engineering, Saveetha Institute of Medical and Technical Sciences (SIMTS), Thandalam, Chennai, Tamil Nadu 602105, India

## Abstract

Candidiasis is a common infection primarily caused by the opportunistic fungus *Candida albicans* *.* Conventional antifungals have been associated with several limitations, and today, antimicrobial photodynamic therapy (aPDT) is raised as an adjuvant treatment. This study aimed to develop a carrier system for the photosensitizer methylene blue (MB) based on a gellan gum (GG) hydrogel, a nontoxic exopolysaccharide. Two formulations consisting of 0.6% and 1.0% (w/v) GG containing MB were prepared and characterized in relation to release kinetics, absorption spectroscopy, and optical shield formation. Then, the effects of MB GG formulations in aPDT using LED irradiation were evaluated against *C. albicans*. For this, aPDT was applied to*C. albicans* in planktonic and biofilm stages and also tested in a *Galleria mellonella* burn infection model. The successful incorporation of MB into the GG hydrogel was confirmed by absorption spectroscopy, with a characteristic peak at 660 nm for intact MB and both GG formulations. The GG0.6% hydrogel released 100% of MB in 12 min, whereas the GG1.0% hydrogel released only 75% of MB after 25 min. Furthermore, no optical shield formation was noticed with the MB gellan formulations in comparison to the aqueous form. In relation to antifungal activity on *C. albicans*, aPDT with GG0.6% and GG1.0% containing MB led to an eradication of planktonic cells and a partial reduction of biofilms, as observed in aPDT with MB aqueous. When aPDT was applied in *G. mellonella* larvae, an increase of 50% and 30% in survival rate was found, respectively, for the groups treated with MB gellan formulation and MB aqueous. In conclusion, the gellan gum formulations designed here were able to release MB and maintain its optical and photodynamic properties against *C. albicans*. In addition, aPDT with MB gellan formulations had higher in vivo efficacy than MB aqueous.

## Graphical abstract

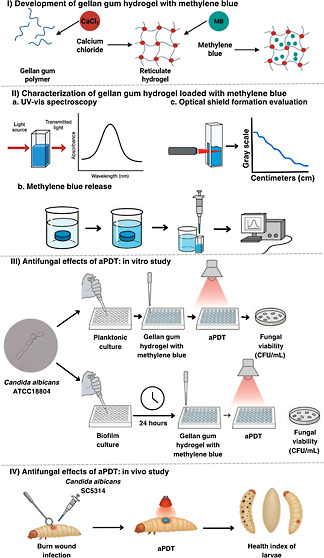

## 1 Introduction

Photodynamic therapy (PDT) is defined as a noninvasive, oxygen-dependent photochemical reaction capable of destroying cells through necrosis or apoptosis, as well as inducing cell damage and killing microorganisms.^1,2^ Its mechanism of action occurs through a light source at a specific wavelength capable of activating the photosensitizer, which, in the presence of oxygen, generates a phototoxic response through reactive oxygen species (ROS).^1,3^ Antimicrobial photodynamic therapy (aPDT) has become an alternative method in the face of the advent of antimicrobial resistance, since oxidative agents act nonspecifically on microorganisms, preventing the development of therapy-resistant strains.^4,5^ However, the effectiveness of this therapy depends on the compatible relationship between microorganisms, types of photosensitizers, light sources, and irradiation conditions.^1^

Among the existing photosensitizers, methylene blue (MB) is widely used in antimicrobial photodynamic therapy and has been approved for clinical use in several countries.^6,7^ MB can be present in monomer or dimer form and shows absorption at wavelengths in the 550–700 nm range, with the monomer having a maximum absorption at 664 nm and the dimer close to 590 nm.^7^ The arrangement of methylene blue directly influences the photochemical mechanism and its respective products in such a way that the monomer produces singlet oxygen through the type II mechanism, while the dimer results in the type I reaction, generating superoxides.^8^ In aPDT, the type II mechanism is more effective in microbial eradication due to the action of singlet oxygen on structures such as DNA, proteins, and lipids, generating oxidative stress.^9^ The absorption spectrum of MB makes PDT compatible with low-power lasers and LEDs that emit light in the visible red range, which are commonly found in healthcare clinics. Given these advantages, methylene blue has been used in the medical and dental fields to treat localized infections caused by bacteria, fungi, viruses, and protozoa.^7,10,11^

In terms of fungal infections, aPDT has been widely studied as a possible therapy for mucosal and cutaneous candidiasis. *Candida* is recognized as a commensal organism, but under favorable conditions, it becomes an opportunistic pathogen to the host.^12^ It expressed several virulence traits that include morphological transition, production of hydrolytic enzymes, and biofilm formation.^13,14^ The biofilm form of *Candida* cells is more tolerant to antifungal agents than planktonic cells. Conversely, the widespread use of antifungals for various purposes has led to resistance to these drugs.^15–17^ As a result, aPDT is considered a potential alternative treatment for candidiasis, since it is minimally invasive and its reactive products act nonspecifically on target cells, eliminating them and preventing the development of resistance to therapy.^18,19^

The use of drug delivery systems has been applied to photosensitizers, which proves to be a promising resource for the application of aPDT in infectious diseases of medical and dental interest. In delivery systems, photosensitizers can be loaded onto different molecules, such as monosaccharides, peptides, nanofibers, low-density lipoproteins (LDLs), inorganic or organic nanoparticles, and polymers such as hydrogels.^20–23^ Recently, gellan gum has been used in experiments with several antibiotics and phytochemicals to enhance its bioavailability and promote its sustained release profile. Gellan gum is an exopolysaccharide produced by the bacterium *Sphingomonas elodea*, which is used in the food industry as a food additive and gelling agent. It is considered a low-cost biomaterial that can be produced on a large scale and is approved by the Food and Drug Administration (FDA) for use in the biomedical field.^24^ The characteristics of gellan gum of interest include its biocompatibility, low toxicity, biodegradability, mucoadhesive properties, and thermos-responsiveness. In the literature, it has been used in research as a carrier for proteins and drugs and in the regeneration of bones and wounds.^25^

In previous studies of our group, gellan gum (GG) hydrogels were used as drug delivery systems targeted for the control of *Candida albicans* infections. GG hydrogels demonstrated the ability to incorporate and release compounds and microorganisms in a controlled manner, such as the polyphenol caffeic acid phenethyl ester (CAPE) and the probiotic *Lactobacillus paracasei* 28.4, highlighting their potential applications for the treatment of candidiasis.^26,27^ In the present study, the GG applications were expanded to aPDT, seeking to extend the retention time of MB at the site of infection and, consequently, to enhance the bioactivity of MB in target cells.^28^ Based on this context, the aim of this study was to develop a methylene blue-loaded GG hydrogel and to evaluate its release profile, optical characteristics, and photodynamic activity against *C. albicans*, using both in vitro assays and an in vivo *Galleria mellonella* burn wound infection model.

## 2 Materials and Methods

### 2.1 Gellan Gum Hydrogel Carrying Methylene Blue

The methylene blue powder (Sigma-Aldrich, SP, Brazil) was prepared in distilled water at a concentration of 1250 μM, followed by sterilization by filtration using a 0.22 μm syringe filter and storage in a dark place. The gellan gum hydrogel (GG) was prepared by dissolving an appropriate (0.6% and 1.0% (w/v)) concentration of powder (Sigma-Aldrich, MO, United States) in distilled water.^26^ The solution was autoclaved at 120 °C for 15 min. After the mixture reached room temperature, aqueous MB was incorporated into the hydrogel under vortex stirring. The carrier system was cross-linked by adding calcium chloride (Dinâmica, SP, Brazil) at a concentration of 1 mM, obtaining a gellan gum hydrogel in both concentrations (0.6% and 1.0%) with MB at a final concentration of 50 μM for the in vitro experiments and 75 μM for the photosensitizer for in vivo experiments.

### 2.2 Light Source

The light source used was an LED device (Irrad-LED, Biopdi, SP, Brazil) consisting of 48 LEDs that emit at a wavelength of 660 nm (visible red). The irradiation parameters were set at an energy density of 30.56 J/cm^2^ and a power density of 42.8 mW/cm^2^. The period of irradiation for planktonic and biofilm was 714 s, and for aPDT in the *G. mellonella* burn model, it was 1051 s.

### 2.3 Characterization of MB-Loaded GG Hydrogels

#### 2.3.1 Absorption Spectroscopy

The absorption spectrum of samples like distilled water, aqueous MB (50 μM), 0.6% and 1.0% (w/v) hydrogels containing distilled water, and 0.6% and 1.0% (w/v) GG hydrogels containing MB was recorded using an Epoch spectrophotometer (BioTek, CA, United States) on a 96-well flat-bottom plate for analysis starting at a length of 400–800 nm, with a reading interval of 1 nm. The results were generated using Gen5 software (BioTek, CA, United States).

#### 2.3.2 Release Kinetics of Methylene Blue from GG Hydrogels

The total immersion method was adopted to study the methylene blue release kinetics of the gellan gum hydrogel. Samples of 1 mL of hydrogels GG0.6% + MB and GG1.0% + MB were transferred to beakers containing 10 mL of distilled water, followed by incubation at 37 °C under 100 rpm agitation in the dark. At predefined times, 2 mL aliquots were collected for reading on an Epoch spectrophotometer (BioTek, CA, United States) at a wavelength of 664 nm. The volume removed from the system was replaced with distilled water after each reading. The total release time was 25 min, with 1 min intervals for graphical construction. The experiment was carried out in triplicate for each hydrogel test.

Based on the λ-max value of the previous experiment, the standard curve of MB was created at the concentrations between 0.7 and 50 μM using the Epoch microplate reader (BioTek, CA, United States). The concentration of MB present in the aliquots was determined by comparing the absorption with the standard curve values.

#### 2.3.3 Optical Shield Formation

The optical shield test consisted of assessing the passage of light through GG hydrogels at concentrations of 0.6% (w/v) (GG0.6% + MB) and 1.0% (w/v) (GG1.0% + MB).^30^ The GG hydrogels containing MB were transferred to a quartz cuvette with four polished sides and an internal width of 10 mm (Kasvi, SP, Brazil) focused in front of a digital camera (EOS Rebel T6, Canon). Subsequently, a laser tip (KP-8008, SP, Brazil) was placed in front of the cuvette, and the images were captured perpendicularly. All of the images of this study were evaluated using ImageJ software (National Institute of Health, USA) in grayscale, and the values obtained per pixel were plotted on a graph of grayscale per distance (cm).

### 2.4 Antifungal Effects of aPDT: In Vitro Study

#### 2.4.1 *Candida albicans* Strain for In Vitro Study

The standard strain of *C. albicans* ATCC 18804 was used in the aPDT study for both planktonic and biofilm assays. The strain was kept frozen in YPD broth with 20% glycerol at −80 °C and activated on Sabouraud dextrose agar (Kasvi, PR, Brazil) at 37 °C for 48 h.

#### 2.4.2 Experimental Groups Performed for aPDT In Vitro Study

The study of photodynamic therapy in planktonic culture and biofilms was divided into the following experimental groups: distilled water in the dark without a photosensitizer and LED irradiation (MB – L−); gellan gum 0.6% with distilled water in the dark (GG0.6 + MB – L−); gellan gum 1.0% with distilled water in the dark (GG1.0 + MB – L−); aqueous methylene blue (50 μM) in the dark (MB + L−); gellan gum 0.6% with methylene blue in the dark (GG0.6 + MB + L−); gellan gum 1.0% with methylene blue in the dark (GG1.0 + MB + L−); PBS irradiated (MB – L+); gellan gum 0.6% with distilled water irradiated (GG0.6 + MB – L+); gellan gum 1.0% with distilled water irradiated (GG1.0 + MB – L+); aqueous methylene blue (50 μM) irradiated (MB + L+); gellan gum 0.6% with methylene blue irradiated (GG0.6 + MB + L+); and gellan gum 1.0% with methylene blue irradiated (GG1.0 + MB + L+). The tests were carried out in 5 repetitions per group (*n* = 5).

#### 2.4.3 *C. albicans* Planktonic Cells

After the microorganism was activated, colonies of *C. albicans* were collected and suspended in sterile PBS solution. The number of cells in the suspension was then counted and standardized by using a mirrored Neubauer chamber (Laboroptik GmbH, Germany). The final concentration of the *C. albicans* suspension was 1 × 10^3^ cells/mL. Photosensitization of the planktonic culture of each suspension was carried out on a 96-well plate, with 100 μL of the standardized suspension and 100 μL of the solution corresponding to the experimental group added to each well. After the plates were prepared, they were wrapped in aluminum foil to avoid exposure to light. The plates remained in the dark under agitation for 15 min during the preirradiation period and were then irradiated by LED according to the parameters previously described.

After irradiation, the contents of the wells were diluted in a 1:10 ratio in dilution microplates (Kasvi, PR, Brazil) containing sterile PBS. Each dilution was seeded using the drop technique on Sabouraud dextrose agar and incubated for 24 h at 37 °C. After the incubation time, the number of colony-forming units was determined in CFU/mL.

#### 2.4.4 *C. albicans* Biofilms

A single colony of *C. albicans* was transferred to a conical tube containing yeast nitrogen base broth (BD Difco, SP, Brazil) with 5% sucrose, followed by incubation for 24 h at 37 °C and 75 rpm. After the incubation period, the suspension was centrifuged at 5000 rpm for 10 min, and the pellet was resuspended in 10 mL of sterile PBS. This procedure was carried out twice. After the contents of the last centrifugation were removed, the cell pellet was resuspended in YNB broth. The suspension was standardized at 1 × 10^7^ cells/mL using a Neubauer chamber and distributed in 96-well flat-bottom plates. The plates were incubated for 24 h at 37 °C under agitation at 75 rpm. After the incubation period, the contents of the wells were aspirated, and the wells were washed twice with sterile PBS.^31^

Then, 200 μL of the test solution was added to the wells according to the experimental groups. Plates were wrapped in aluminum foil and placed on an orbital shaker for 15 min (preirradiation), followed by LED irradiation according to the parameters previously described. After the treatment, the wells were washed with sterile PBS. The contents of each well were removed from the plate using an ultrasonic homogenizer with a power of 7 W for 30 s. The contents of the wells were diluted in a ratio of 1:10 in dilution microplates containing a PBS solution. Each dilution was seeded using the drop technique on Sabouraud dextrose agar and incubated for 24 h at 37 °C. After the incubation, the number of colony-forming units was determined and expressed as CFU/mL.

### 2.5 Effects of aPDT in a Burn Model in *G. mellonella*: In Vivo Study

#### 2.5.1 *G. mellonella* Larvae

The *G. mellonella* larvae kept at the Invertebrate Laboratory of the Institute of Science and Technology/UNESP were used without visible signs of disease. In a previous experiment, the best concentration of gellan gum used was defined. To do this, a survival curve was drawn for the larvae over a 120 h period after burning and infection, followed by treatment according to the experimental group. Each experimental group was made up of 10 randomly selected larvae with a body weight between 250 and 300 mg, light-colored and free of spots on their cuticle. All analyses were carried out in duplicate, and the larvae were not fed during the experiment. For each trial, a control group was always included, made up of larvae that did not receive any intervention, to control the quality of the larvae rearing.^32,33^

#### 2.5.2 *C. albicans* Strain for In Vivo Study

For the burn infection model in *G. mellonella*, the *C. albicans* SC5314 strain was employed, as indicated by Terra Garcia et al. (2025).^34^ This strain presents ROB1 heterozygotes in dominant allele form, resulting in an increased capacity for filamentation and tissue invasion, which favor the development of infection on burned tissue.^29^ From frozen stocks, the strain was activated on Sabouraud dextrose agar at 37 °C for 48 h.

#### 2.5.3 Burn Induction, Infection, and aPDT

The burn model was adopted from the following literature.^32^ To prevent excessive movement and facilitate easy handling, the larvae were placed in sterile Petri dishes and kept in a refrigerator for approximately 20 min. The cuticle of the larvae was cleaned with 70% ethanol. Subsequently, the burn lesion was induced with a heated steel loop to reach an area of approximately 2 mm. The loop used was heated to a high temperature, and it was applied to the dorsal portion of the larvae for a fixed period of 4 s. Immediately after the burn, 10 μL of the *C. albicans* suspension containing 1 × 10^9^ cells/mL were applied three times to the site of the formed lesion, with intervals of 15 min between them. After a 30 min interval from the last application of the suspension, the larvae were given 10 μL of GG with MB or aqueous MB on the burn wound. After 30 min in the dark, the larvae were irradiated according to the parameters previously described.

#### 2.5.4 Experimental Groups Performed for aPDT In Vivo Study

In the *G. mellonella* model to select the best hydrogel concentration to be used, an initial test was carried out, consisting of the following groups: control without manipulation; burn without infection; burn with infection without treatment; burn with infection aPDT with gellan gum 0.6% with MB (75 μM final concentration); and burn with infection aPDT with gellan gum 1.0% with MB (75 μM final concentration). To select the ideal concentration of MB in gellan gum at 0.6%, a test was performed by the following groups in a survival curve during 120 h: control without manipulation; burn without infection; burn with infection without treatment; and burn with infection treated by aPDT with GG 0.6% carrying MB at 50, 75, 150, and 300 μM (final concentrations).

After the best concentration was selected, the study was carried out with the following groups: healthy larvae not treated; burn not treated; burn infection not treated; burn infection treated with light; burn infection treated with MB (75 μM final concentration) in the dark; burn infection treated with GG 0.6 in the dark; burn infection treated with aPDT using aqueous MB (75 μM final concentration); and burn infection treated with aPDT using GG 0.6% carrying MB (75 μM final concentration).

Each group consisted of 10 larvae, and each analysis was carried out in duplicate.

#### 2.5.5 Larval Survival Curve

After aPDT, the larvae were placed in 24-well plates, incubated at 37 °C in the dark, and analyzed daily for the course of 120 h (5 days). The number of dead larvae was recorded daily to calculate the survival curve. Larvae that did not show any movement when touched were considered dead and were removed from the groups as soon as they were identified.

#### 2.5.6 Health Score of Larvae

The larvae were monitored according to a pathological scoring system proposed by literature^34^ for the following attributes: movement activity, extent of silk production, melanization, and survival production (cocoon formation). The scheme of the health index experiment evaluating *G. mellonella* signals of health and groups of analysis is shown in Figure 1.

**Figure 1:**
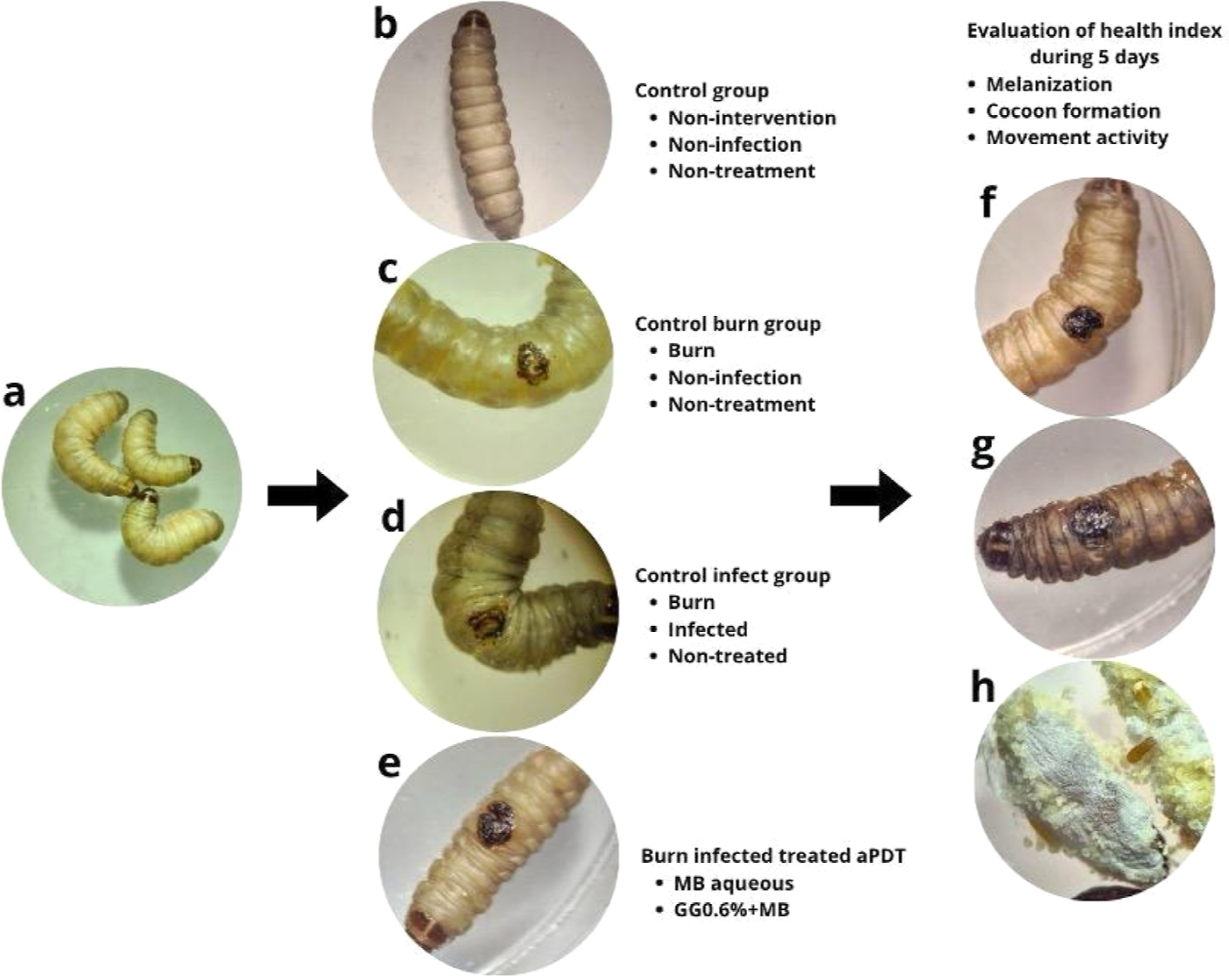
Scheme of the health index experiment evaluating *Galleria mellonella* signals of health and respective groups of analysis. (a) Healthy selected larvae of *G. mellonella* without signals of disease (no melanization), normal movement, and cocoon production; (b) control group composed of larvae with no burn, no infection, and no treatment; (c) burn control group representing larvae that received only a burn but were not infected or treated; (d) burn followed by infection at the cutis of *G. mellonella* without treatment; (e) group testing the efficacy of aPDT mediated by MB aqueous solution and GG0.6% + MB; (f) example of a larva without melanization; (g) example of a larva with melanization; (h) example of cocoon formation by the larvae.

A score was given for each attribute. Movement activity: 0—no movement; 1—minimal movement with stimulation; 2—movement with stimulation; and 3—movement without stimulation. Cocoon formation: 0—no cocoon; 0.5—incomplete; and 1 complete cocoon. Melanization: 0—complete; 1—dark spots on the brown larva; 2—more than three dots on the beige larva; 3—less than three dots on the beige larva; and 4—no melanization. Survival: 0—dead larva and 2—alive. The sum of the points corresponded to a general index of the larva’s health. Healthy larvae were scored between 9 and 10, while dead larvae received a score of 0. The average scores obtained for each attribute analyzed were transformed into percentages of 100% and are represented graphically.

### 2.6 Statistical Analysis

The results of aPDT in planktonic and biofilm experiments were evaluated by Analysis of Variance (ANOVA) and Dunnett’s test, comparing the intervention groups with the control group (MB – L−). The data obtained from the health index of the *G. mellonella* larvae were analyzed by the Friedman test followed by Dunn’s test. The Kaplan–Meier method was used for the survival curve tests in *G. mellonella*, with the significance level calculated using the log-rank test (Mantel–Cox). The GraphPad Prism 5.0 program was used for all the experimental tests with a significance level of 5%.

## 3 Results

### 3.1 Confirmation of Incorporation of MB in GG Using Absorption Spectroscopy

The absorbance spectra were recorded for the following samples: distilled water, gellan gum at concentrations of 0.6% and 1.0% with distilled water, gellan gum at concentrations of 0.6% and 1.0% with added methylene blue, and aqueous methylene blue (50 μM) (Figure 2). The groups containing MB showed two strong absorption bands at 600 and 660 nm. The addition of MB to the 0.6% and 1.0% GG hydrogels did not affect the absorption peak of the photosensitizer, which remained at 660 nm. The spectroscopy graph visually indicates a higher proportion of MB in aggregated monomer form (λ = 660 nm) compared to dimer (λ = 610 nm), both in the aqueous form of the photosensitizer and in the 0.6% and 1.0% hydrogels.

**Figure 2:**
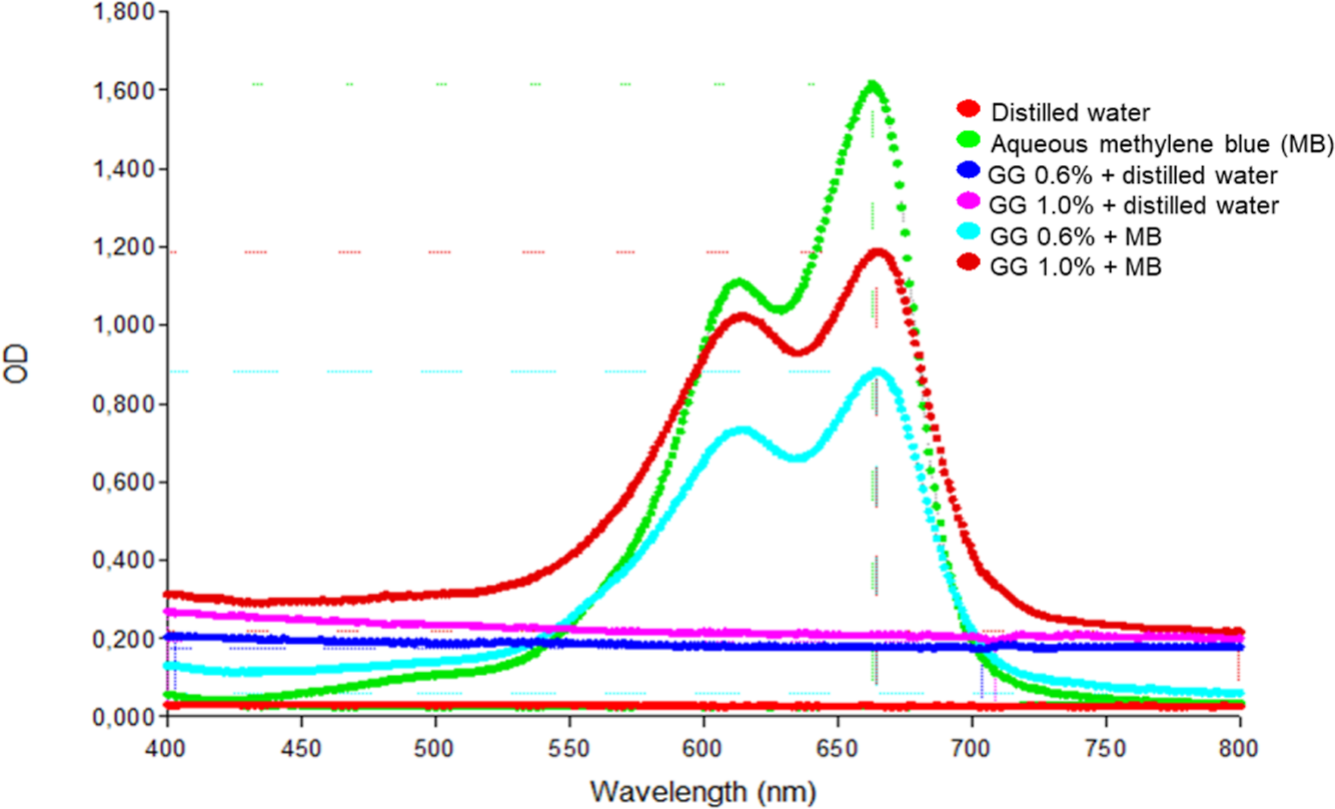
Spectroscopic analysis in the UV–visible region. Axis *Y* refers to the optical density (OD) of light absorption. Axis *X* is the wavelength of emitted light by the equipment in nanometers (nm). Aqueous methylene blue (MB) has the maximum optical density (OD max: 1.600), followed by gellan gum hydrogel 1.0% (w/v) with methylene blue (GG1.0% + MB) (OD max: 1.200) and hydrogel 0.6% (w/v) with methylene blue (GG0.6% + MB) (OD max: 0.900). Gellan gum hydrogel 0.6% and 1.0% (w/v) with distilled water kept constant the OD at 0.200 and 0.300 values, respectively. Constant scanning at 0.300 refers to an empty well and distilled water.

The aqueous form of the photosensitizer exhibited the highest optical density (1.600), followed by the 1.0% (w/v) hydrogel containing the photosensitizer, which had an OD of 1.200, and the 0.6% (w/v) hydrogel with methylene blue, which recorded an OD of 0.900. Distilled water, 1.0% (w/v) and 0.6% (w/v) hydrogels without a photosensitizer, and an empty well were used as controls to check for possible interference in the reading of the material’s maximum OD values. It was observed that the hydrogels with distilled water obtained a constant reading in the range of 0.300 optical density and that the distilled water and empty well obtained a reading close to zero OD.

### 3.2 Methylene Blue Released from the System Carrier

The standard curve for methylene blue was described by the equation *y* = 0.0171*x* + 0.0921, where “*y*” represents absorbance and “*x*” denotes the concentration of methylene blue in μM. The coefficient of determination (*R* ^2^) for the regression was 0.9982. The graph of methylene blue release from GG, presented in Figure 3, was generated based on the photosensitizer’s standard curve. This enabled the calculation of methylene blue concentrations released from the 0.6% and 1.0% (w/v) GG hydrogels into the reaction medium over the 0-to-25 min interval.

**Figure 3:**
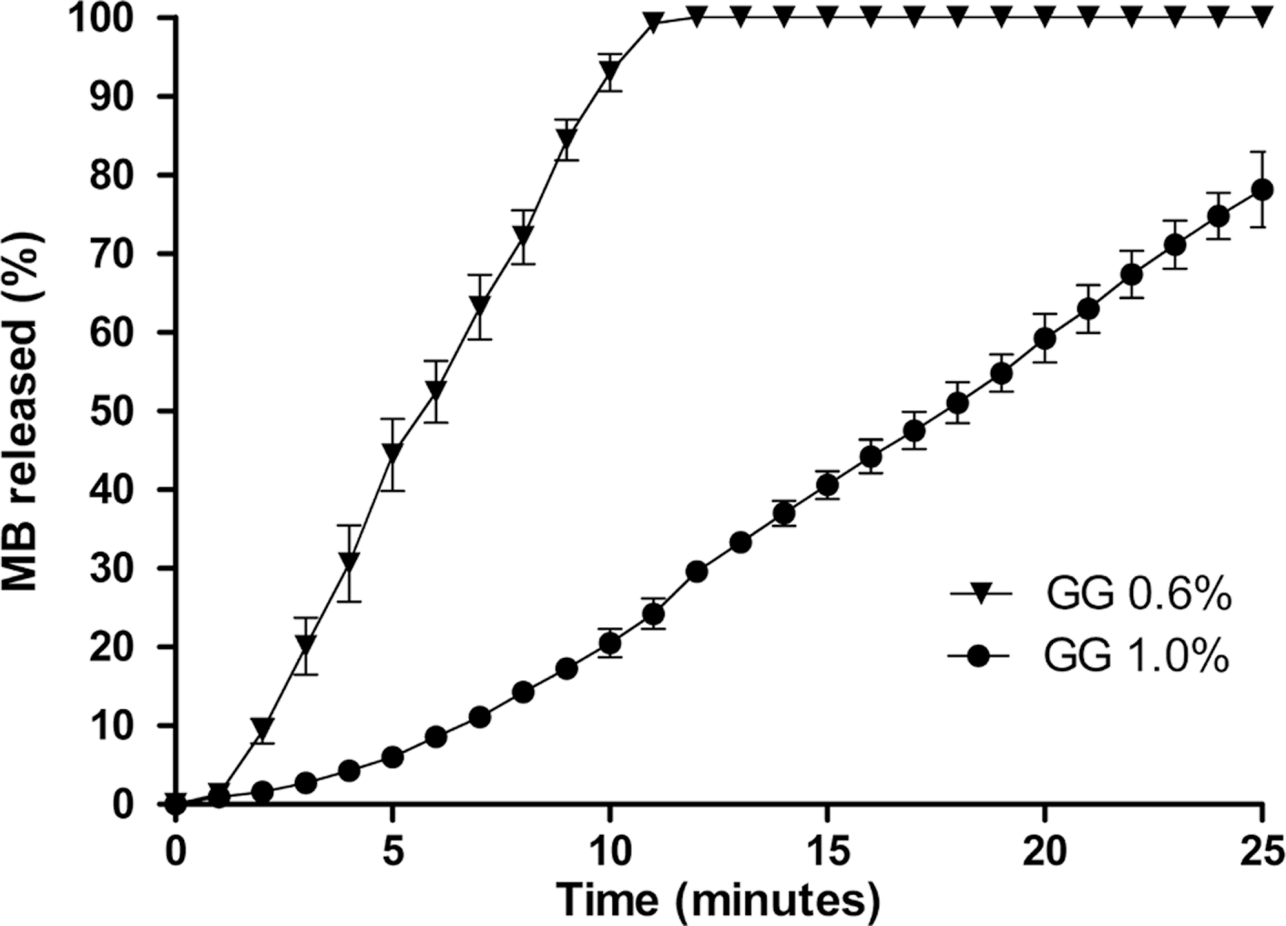
Percentage of methylene blue (MB) released by time (minute). The graph shows the released profile of the photosensitizer during the time of 25 min. Each point represents the percentage by minute of methylene blue released from the groups “GG 0.6%” and “GG 1.0%”.

The fastest release was observed for the 0.6% hydrogel (w/v), reaching 100% release in 10 min. For the same experimental period, the 1.0% (w/v) hydrogel resulted in a smaller release, approximately 75% of the total, as well as a longer exposure time to the reaction medium to obtain this release. The results indicated an indirect relationship between the concentration of GG and the release capacity of the photosensitizer.

### 3.3 Analysis of Optical Shield Formation

The laser showed success in passing through the 1 cm optical path for the MB formulations at concentrations of 0.6% and 1.0% GG, similar to the aqueous MB solution. The results were obtained by visual analysis and quantitative analysis by graphical representation (Figure 4). The light intensity per pixel is proportional to the value on the gray scale. In both media, there was a drop in the gray value as the light beam traveled through the cuvette. MB in aqueous form (Figure 4C) showed greater homogeneity in the optical path compared to the 0.6% (w/v) hydrogels (Figure 4A) and 1.0% (w/v) hydrogels (Figure 4B). At the end of the graphs, there is an increase in the gray value, which coincides with the area at the end of the cuvette that has reflected the light. Despite these small variations, the three media allowed the laser to pass through the entire optical path.

**Figure 4:**
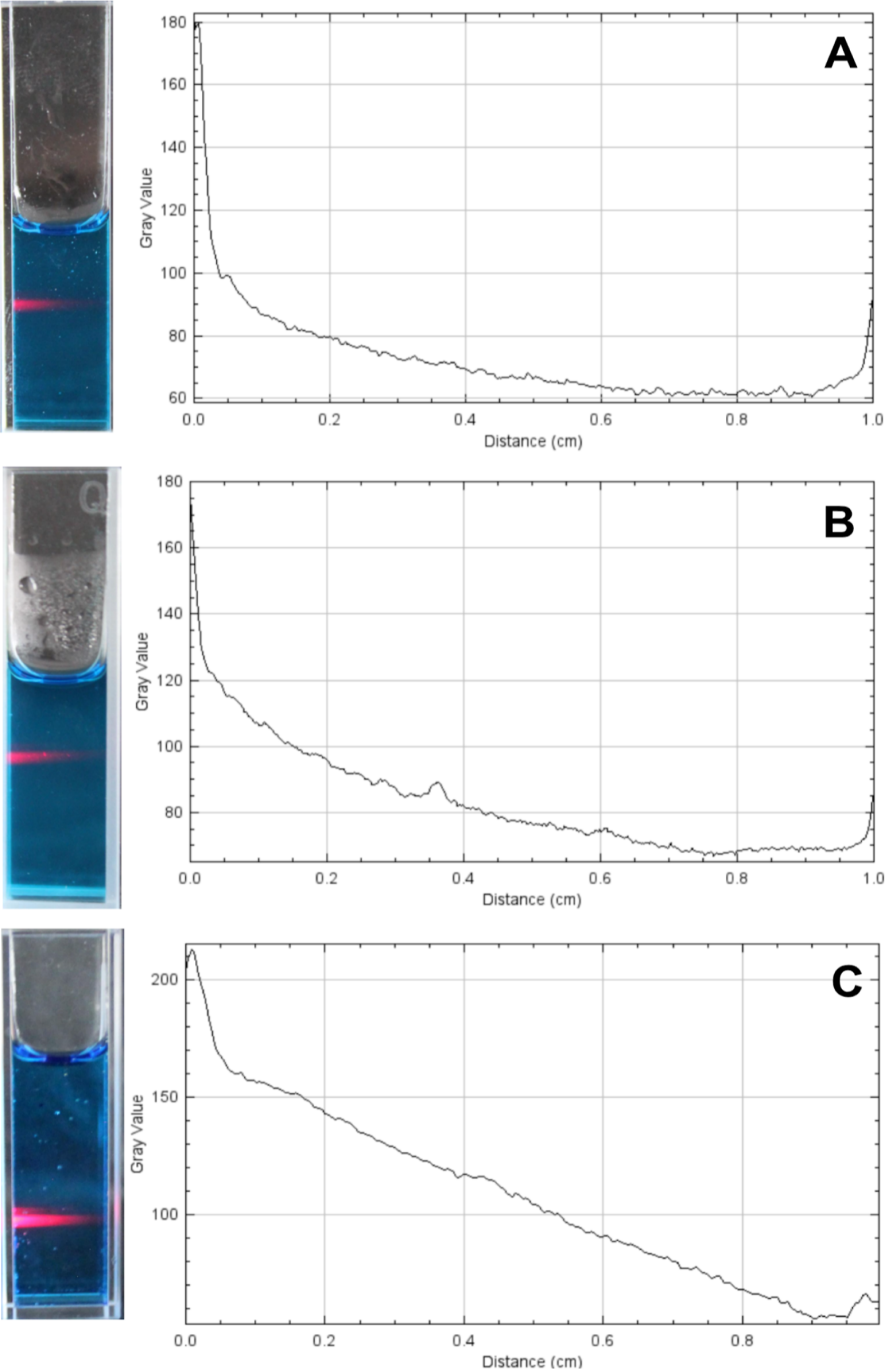
Analysis of the optical shield test to analyze the passage of light through methylene blue formulations and quantitative analysis by graphical representation of gray scale from optical shield with gray value per pixel (*Y* axis) and distance in centimeters (*X* axis): (A) 0.6% (w/v) GG hydrogel containing MB; (B) 1.0% (w/v) GG hydrogel containing MB; and (C) aqueous MB.

### 3.4 Effects of aPDT on Planktonic Cells of *C. albicans*

The results of aPDT on planktonic cultures are shown in Figure 5. The control group without light exposure (MB – L) showed *C. albicans* fungal loads of 3.5 Log_10_, as did the 0.6% (GG0.6 + MB – L−) and 1.0% (GG1.0 + MB – L−) groups in the absence of light, demonstrating that photosensitizer-free gellan gum did not result in antimicrobial action. In the same way, the groups treated with aqueous methylene blue (MB + L−), gellan gum 0.6% (GG0.6 + MB + L−) and gellan gum 1.0% (GG1.0 + MB + L−), both of which contained a photosensitizer in the absence of light, showed growth similar to that of the control group, resulting in 3.5 Log_10_. This suggests that the presence of a photosensitizer in the dark did not exert any cytotoxic action on the planktonic culture. Similar results (3.5 Log_10_) were observed for the groups exposed to irradiation without photosensitizer, including the irradiation control (MB – L+), 0.6% gellan gum (GG0.6 + MB – L+), and 1.0% gellan gum (GG1.0 + MB – L+). This indicates that LED irradiation, in the absence of a photosensitizer, was ineffective in reducing yeast cell viability.

**Figure 5:**
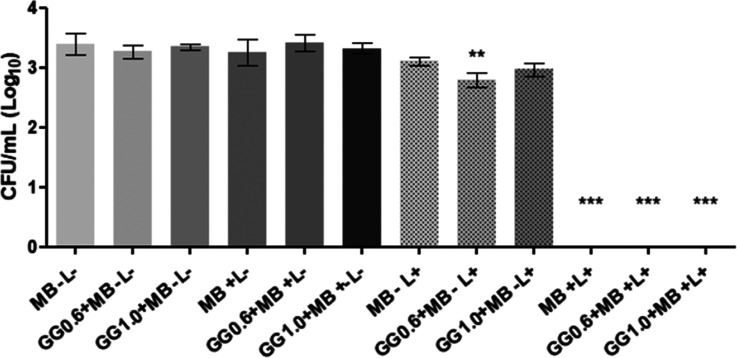
Effects of photodynamic therapy on planktonic cultures of *C. albicans* with the mean and standard deviation of the data in CFU/mL obtained in the photodynamic therapy test on planktonic cultures of *C. albicans*. Distilled water in the dark without a photosensitizer and LED irradiation (MB – L−); gellan gum 0.6% with distilled water in the dark (GG 0.6 + MB – L−); gellan gum 1.0% with distilled water in the dark (GG1.0 + MB – L−); aqueous methylene blue (50 μM) in the dark (MB + L−); gellan gum 0.6% with methylene blue in the dark (GG0.6 + MB + L−); gellan gum 1.0% with methylene blue in the dark (GG1.0 + MB + L−); PBS irradiated (MB – L+); gellan gum 0.6% with distilled water irradiated (GG0.6 + MB – L+); gellan gum 1.0% with distilled water irradiated (GG1.0 + MB – L+); aqueous methylene blue (50 μM) irradiated (MB + L+); gellan gum 0.6% with methylene blue irradiated (GG0.6 + MB + L+); and gellan gum 1.0% with methylene blue irradiated (GG1.0 + MB + L+). Groups with statistically significant differences in the Dunnett’s multiple comparisons are represented by different symbols (**p < 0.01 and ***p < 0.001).

Promisingly, aPDT groups mediated by 50 μM aqueous methylene blue (MB + L+), 0.6% gellan gum with methylene blue (GG0.6 + MB + L+), and 1.0% gellan gum with methylene blue (GG1.0 + MB + L+) resulted in a total and significant reduction (*p* < 0.05) in planktonic culture. These data demonstrated the antifungal action of photodynamic therapy in *C. albicans* planktonic cells as well as demonstrated that the 0.6% (w/v) and 1.0% (w/v) gellan gum hydrogels were able to maintain photodynamic properties like those of the aqueous photosensitizer methylene blue.

### 3.5 Effects of aPDT in *C. albicans* Biofilms

The results of aPDT on the *Candida* biofilm are shown in Figure 6. In the control group (MB – L−) as well as 0.6% gellan gum (GG0.6 + MB – L−) and 1.0% gellan gum (GG1.0 + MB – L−) a growth of 7 Log_10_ was recorded. Therefore, it confirmed that both formulations without MB had no effect on the biofilm formed. The groups that contained methylene blue in the absence of light, such as aqueous methylene blue (MB + L−), gellan gum 0.6% (w/v) with methylene blue (GG0.6 + MB + L−), and gellan gum 1.0% (w/v) with methylene blue (GG1.0 + MB + L−), also resulted in 7 Log_10_. This result showed that the presence of methylene blue in the dark was unable to reduce the microbial load of the biofilm, and the incorporation of the photosensitizer into the gellan gum carrier system had no cytotoxic action in the dark. The groups irradiated in the absence of the photosensitizer methylene blue, the light control group (MB – L+), and the gellan gum 1.0% (m/v) group (GG1.0 + MB – L+) maintained a 7 Log_10_ count similar to the control (MB – L−). On the other hand, photodynamic therapy groups mediated by aqueous methylene blue (MB + L+), gellan gum 0.6% (w/v) (GG0.6 + MB + L+), and gellan gum 1.0% (w/v) (GG1.0 + MB + L+) showed significant reductions of 1.5 Log_10_, 1.0 Log_10_, and 1.0 Log_10_, respectively.

**Figure 6:**
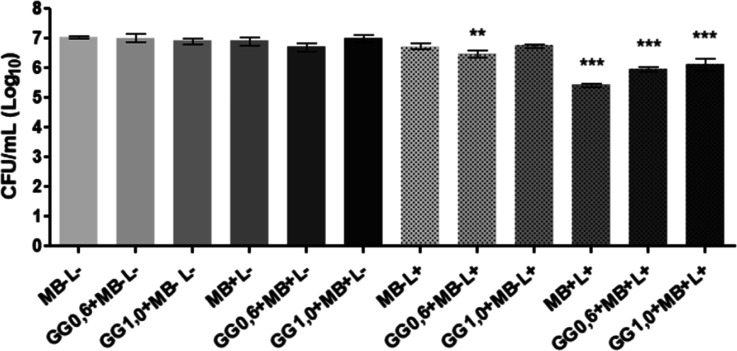
Effects of photodynamic therapy on the biofilm of *C. albicans* with the mean and standard deviation of the data in CFU/mL obtained in the photodynamic therapy test on the biofilm of *C. albicans*. Distilled water in the dark without a photosensitizer and LED irradiation (MB – L−); gellan gum 0.6% with distilled water in the dark (GG0.6 + MB – L−); gellan gum 1.0% with distilled water in the dark (GG1.0 + MB – L−); aqueous methylene blue (50 μM) in the dark (MB + L−); gellan gum 0.6% with methylene blue in the dark (GG0.6 + MB + L−); gellan gum 1.0% with methylene blue in the dark (GG1.0 + MB + L−); PBS irradiated (MB – L+); gellan gum 0.6% with distilled water irradiated (GG0.6 + MB – L+); gellan gum 1.0% with distilled water irradiated (GG1.0 + MB – L+); aqueous methylene blue (50 μM) irradiated (MB + L+); gellan gum 0.6% with methylene blue irradiated (GG0.6 + MB + L+); and gellan gum 1.0% with methylene blue irradiated (GG1.0 + MB + L+). Groups with statistically significant differences in the Dunnett’s multiple comparisons test are represented by different symbols (**p < 0.01 and ***p < 0.001).

### 3.6 Effects of aPDT in the *G. mellonella* Burn Model

#### 3.6.1 Survival Curve

Groups of burn-infected larvae treated with aPDT mediated by GG0.6% + MB (75 μM) and GG1% + MB (75 μM) showed an increase in larval survival of 50% (*p* = 0.0497) and 40% (*p* = 0.1245), respectively, compared to the burn infection not-treated group (Figure 7A). Due to its greater survival capacity, the GG0.6% + MB hydrogel was selected to continue the in vivo study.

**Figure 7:**
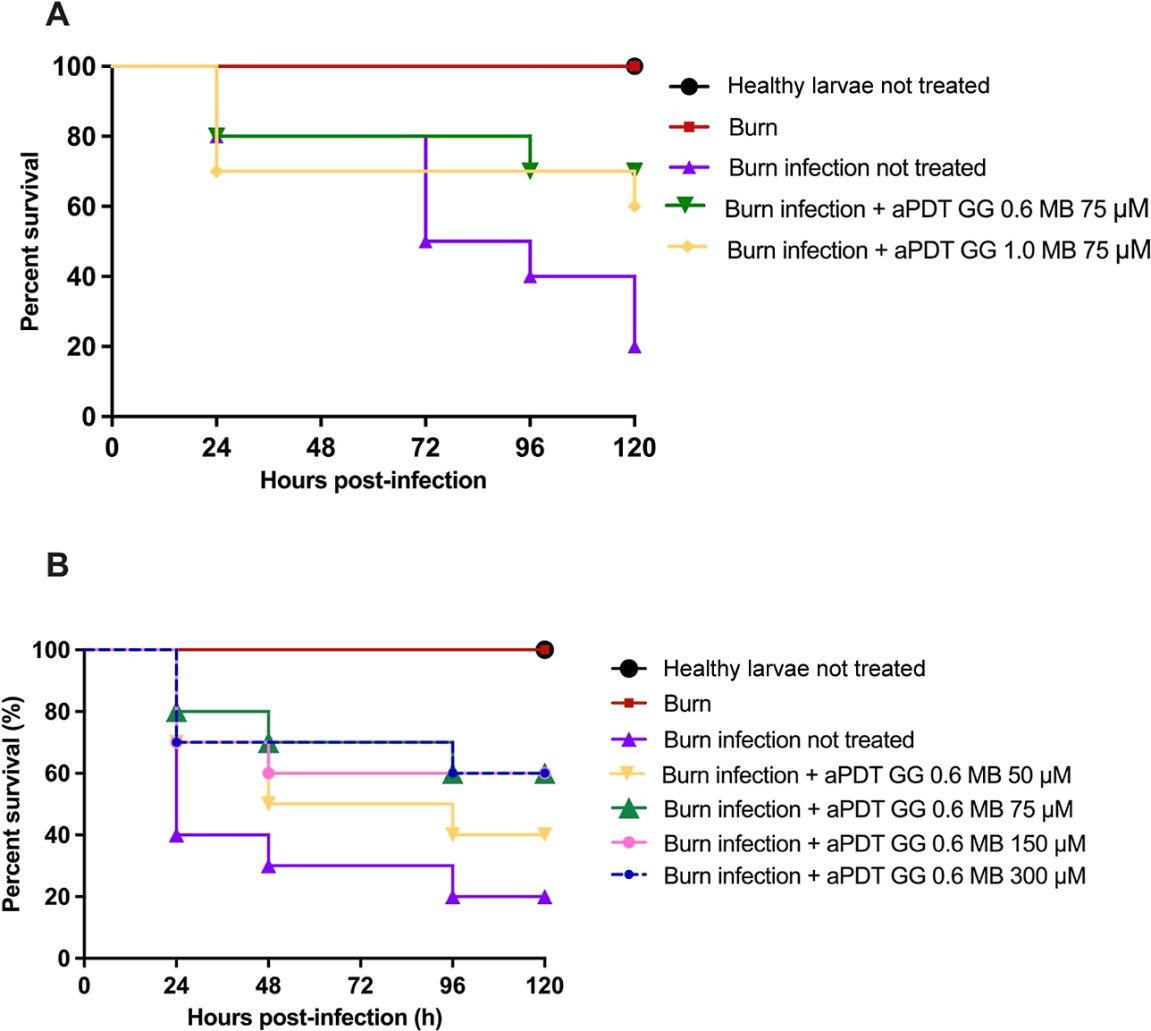
(A) Initial survival curve indicating the percentage of larval survival after 120 h. Groups: healthy larvae; burn; burn with untreated infection; burn treated with aPDT GG0.6% + MB; burn treated with aPDT GG1.0% + MB; (B) survival curve indicating the percentage of larval survival after 120 h of treatment with different concentrations of methylene blue in GG0.6%. Groups: healthy larvae; burn; burn with untreated infection; burn treated with aPDT GG0.6% + MB at 50 μM, 75 μM, 150 μM, and 300 μM.

Continuing the survival curve analysis of the MB concentration (Figure 7B), it was observed that larvae in the burn and infection group without treatment showed a survival rate of 20% at the end of the experiment. Groups treated with aPDT mediated by GG 0.6% containing MB at 50 μM demonstrated 40% survival. In contrast, groups treated with aPDT mediated by GG 0.6% containing MB at concentrations of 75, 150, and 300 μM exhibited a survival rate of 60%. Compared to the untreated control group, statistically significant differences were observed for the GG0.6% with MB 50 μM group (*p* = 0.2697), GG0.6% with MB 75 μM group (*p* = 0.0557), GG 0.6% with MB 150 μM group (*p* = 0.0800), and GG0.6% with MB 300 μM group (*p* = 0.0663). These results suggest that MB concentrations above 75 μM did not enhance the aPDT effect. Based on these findings, the *G. mellonella* health index experiments were carried out using the concentration of 75 μM in the GG0.6% hydrogel.

Next, a more detailed survival curve assay was performed involving additional experimental groups (Figure 8). The larvae in the control group, which received no manipulation and only burning, had survival rates of 100% and 90%, respectively. The survival rates of larvae in treatment groups were compared with the untreated groups. The group treated only with irradiation promoted a 20% improvement in larval survival (*p* = 0.3921). There were no significant differences noted among the groups of free MB (*p* = 0.7967) and GG0.6% + MB without irradiation (*p* = 0.7093). In contrast, larvae with burns treated with aPDT mediated by GG0.6% + MB (*p* = 0.0331) and aqueous MB (*p* = 0.0669) showed an increase in larval survival of 50% and 30%, respectively.

**Figure 8:**
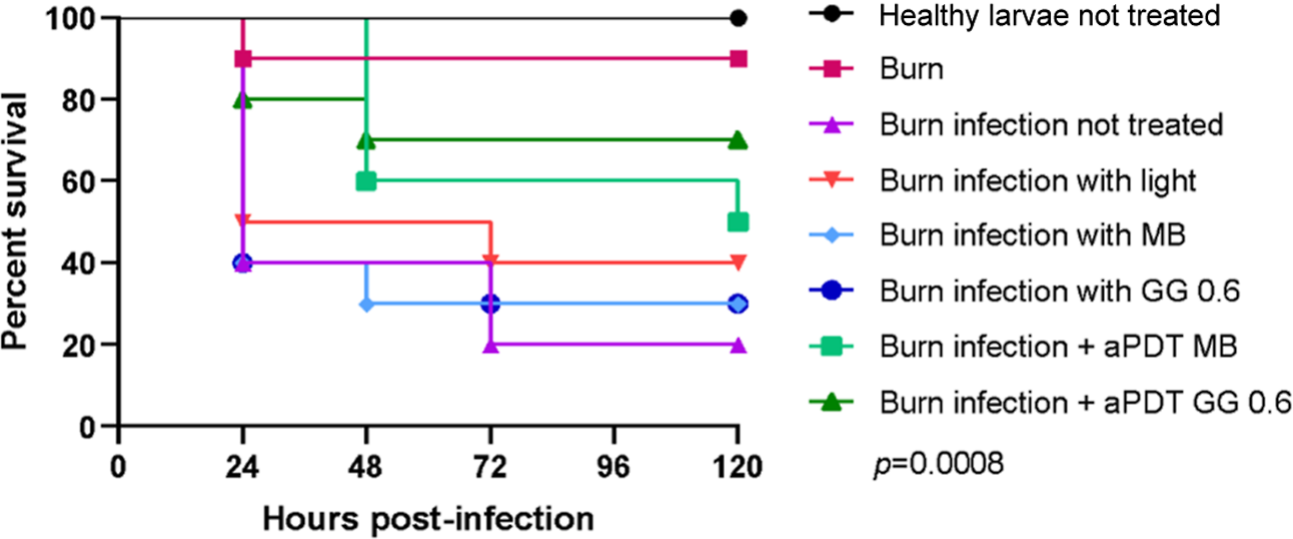
Survival curve of *G. mellonella* infected with *C. albicans* with aPDT mediated by GG0.6% + MB in relation to burn infection not treated (*p* = 0.0331).

#### 3.6.2 Larval Health Index

Regarding the larval health index, the control group without infection showed 100% larval movement activity (Figure 9a), while the group with burns and infections but without treatment had 20% activity. Larvae exposed to aPDT with free MB maintained 50% movement activity compared to the group with infection without treatment. In contrast, larvae treatment with 0.6% GG with MB in aPDT exhibited around 66% movement activity. The cocoon formation is the second most common parameter considered for analysis; the results are presented in Figure 9b. The healthy larvae group had an 80% cocoon formation rate, whereas all the groups with burn induction, independently of the type of treatment, showed only 5% cocoon formation.

**Figure 9:**
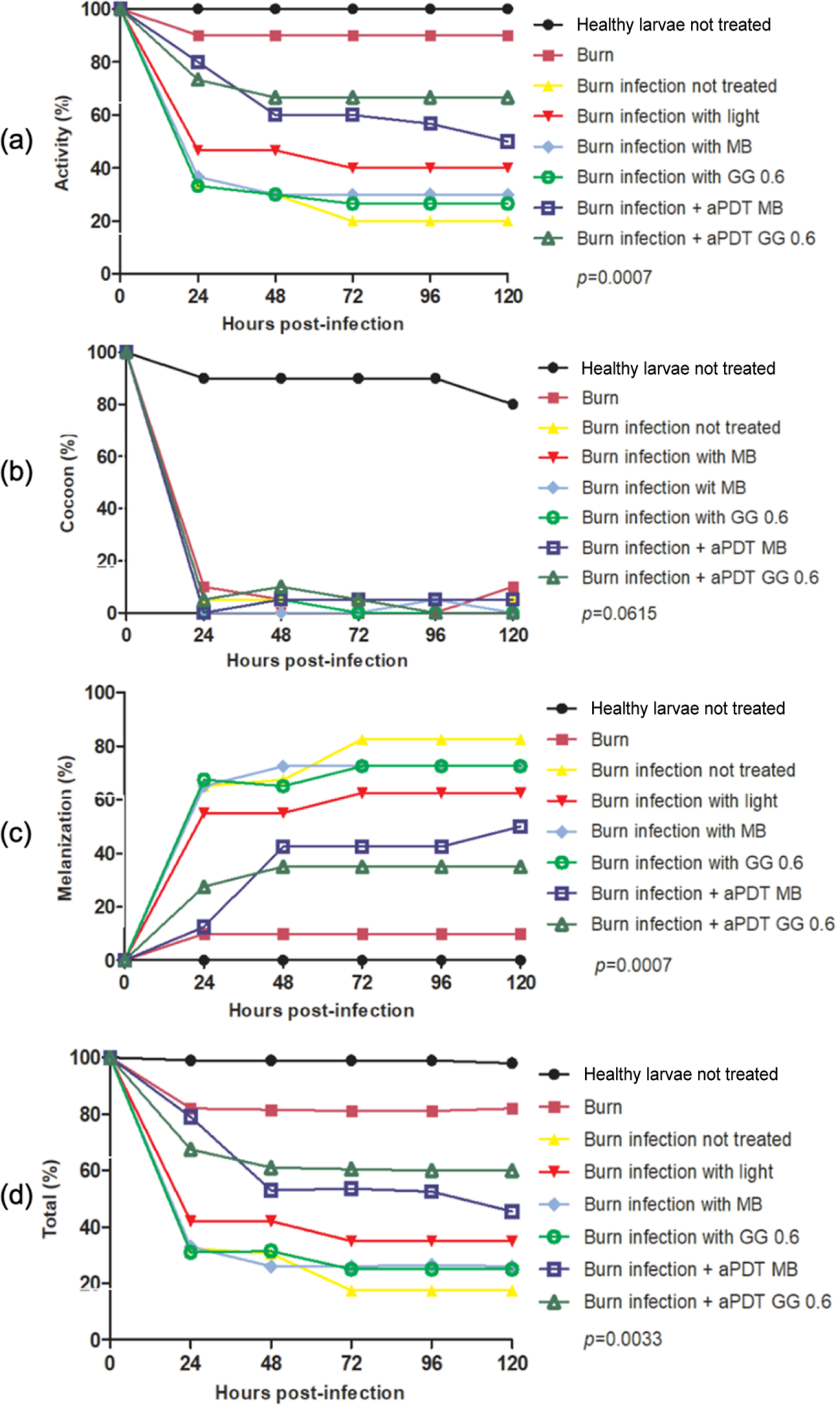
Means of scores obtained in the health index analysis. Friedman and Dunn’s test (*p* ≤ 0.05). The “Burn infection not treated” group was compared to the “Burn infection + aPDT MB” and “Burn infection + aPDT GG 0.6” groups in relation to (a) movement activity, (b) cocoon formation, (c) melanization, and (d) total score.

Melanization is the key component of the larvae immune system, where the insect produces melanin in response to surrounding microorganisms, making it a critical indicator of larvae health status. The rate of melanization during the experimentation is presented in Figure 9c. The larvae in the burn and infection group without treatment showed a melanization index of 82.5%, while the control group had an index of 0%. However, the rate of melanization was consistently reduced in the groups treated with aPDT mediated by aqueous MB (32.5%) and GG0.6% + MB (47.5%). Finally, concerning the overall health score (Figure 9d), the group treated with aPDT mediated by aqueous MB showed a 28% increase, and the group treated with 0.6% + GG demonstrated a 42.5% increase, both compared to the untreated infected group.

## 4 Discussion

To increase the application of aPDT in treating *C. albicans* infections, the photosensitizer MB was incorporated into the GG hydrogel system, and its optical, release, photodynamic, and antimicrobial properties were tested.

In relation to the analysis of optical properties, as reported in the literature,^36^ our results confirmed that methylene blue in aqueous solution has an absorption range between 600 and 660 nm, with a maximum absorption peak at 660 nm. When incorporated into the GG hydrogel, MB retained this absorption peak at 660 nm, indicating that its optical properties were preserved within the hydrogel matrix. The presence of this peak in the 660 nm region, associated with the monomer area, in both the hydrogel and aqueous MB (50 μM) solutions suggests a predominance of the type II photodynamic reaction during photodynamic therapy.^7^ Although the excitation peak of methylene blue remained at 660 nm, variations in the optical densities (OD) were observed in solutions containing different concentrations of gellan gum. With the same concentration of MB (50 μM), the absorption of the 0.1% GG formulation was higher than 0.6% GG, suggesting possible interactions of MB with the hydrogel matrix. These results highlight the relevance of this study as an exploratory step in characterizing this system. However, further experiments are required to clarify the possible interaction between methylene blue and gellan gum as well as their effects on the photosensitizer absorbance.

In the optical shield test, it was observed that all groups containing methylene blue (50 μM), either alone or combined with 0.6% and 1.0% gellan gum (w/v), exhibited a reduction in light transmission through the 1 cm cuvette. Despite this gradual decrease, all groups demonstrated similar light transmission, indicating that gellan gum did not hinder the light beam from passing through the optical path. In a related study,^30^ the authors investigated the visual interference of various concentrations of methylene blue in an aqueous medium on laser beam transmission. They found that concentrations of 50 μM and 100 μM allowed complete light passage through the optical path, while 150 μM permitted approximately half the light to pass, and at 300 μM, the beam was restricted to the beginning of the optical path.^30^ In our study, both formulated and aqueous systems had a final concentration of methylene blue of 50 μM, which permitted a greater proportion of light transmission. Therefore, gellan gum acted as a conventional diluent of the photosensitizer without causing an optical shielding phenomenon.

Gellan gum successfully incorporated photosensitizer MB and promoted its controlled release. Our results showed a faster release of the photosensitizer from the 0.6% (w/v) GG formulation compared to the 1.0% (w/v) hydrogel. The relation between an increase in the concentration of gellan gum in the formulation and a consequent reduction in the release of compounds was also observed in the literature. Garcia et al. observed a similar profile of the release of caffeic acid phenethyl ester (CAPE) from hydrogels of gellan gum. When in lower concentrations, GG promoted quick release of CAPE compared to higher concentrations.^27^ Due to the cross-linked polymer structure of gellan gum formed by adding calcium chloride, the tridimensional structure can create spaces where the photosensitizer gets trapped. Consequently, the higher the concentration of these hydrogel structures, the lower will be the release of methylene blue.^37,38^ This could explain why not all of the photosensitizer was released from the GG hydrogel at a concentration of 1.0% during the evaluation time (25 min).

The capacity of controlled release of gellan gum observed in this study can be comparable to other delivery systems reported in the literature, including chitosan–gellan gum hydrogels, liposomal formulations, and conventional liposomes.^39–42^ Zheng et al. developed a mixed gellan gum and chitosan hydrogel system to address the limitations of Pickering emulsions for some gastrointestinal diseases. Using Nile red dye as a release model, the authors found a higher percentage of release from the 0.2% formulation compared to the 0.8% formulation over the same analysis period.^39^ Some researchers investigated the liposomal formulations as a delivery system of photosensitizers for aPDT. De Leo et al. employed polydopamine-coated liposomes for the delivery of methylene blue (MB). The cumulative release of MB from these liposomes was assessed using a dialysis-based method. In the first 4 h, a rapid release of MB was observed, followed by a slower release over the next 7 h, reaching a total of 45% or more.^40^ Similarly, Soares Lima et al. explored poly-ε-caprolactone-based liposomes for MB delivery, showing an average release of 85.88% within the first hour, with a slight delay in release observed after 5 h.^41^ Earlier research by Wu et al. also supported these findings, showing a rapid release of approximately 95% of MB within 8 h when loaded into zwitterionic polymer-based liposomes.^42^ Taken together, these studies confirm that delivery systems based on liposomes or hydrogels like gellan gum can provide a controlled release of MB; however, the release profile depends on the concentration of methylene blue and the type and concentration of the drug carrier. In addition, the release time can become faster in clinical applications by contact with biological fluids. For example, it was reported that gellan gum can be degraded by the enzymatic activity of lysozyme present in human saliva.^43^

Another important point to be considered in relation to photosensitizers released from delivery systems is the preirradiation time. According to the review article,^44^ the preirradiation time in antimicrobial photodynamic therapy experiments ranges from 1 to 30 min, with 5 min being the most commonly used. In our study, we employed a preirradiation time of 15 min for the in vitro experiments and 30 min for the in vivo experiments, both falling within the commonly reported range. These preirradiation times were sufficient to achieve the release of 100% of methylene blue (MB) from GG0.6% and 75% of the photosensitizer from GG1.0%, thus meeting the objective of this study: to promote controlled release to provide an efficient therapeutic approach for future applications.

In view of these results, methylene blue incorporated into 0.6% and 1% gellan gum formulations was tested in photodynamic therapy against *C. albicans* in planktonic cultures. Due to the lack of a specific document for standardizing microbial suspensions for antimicrobial susceptibility testing in photodynamic therapy, the standard suspension for the test in planktonic culture was adapted from Standard M27-A2 drawn by consensus of the Clinical and Laboratory Standards Institute (CLSI).^45^ This aims to standardize the test microbial suspension at a final concentration of 5.0 × 10^2^ to 2.5 × 10^3^ cells per milliliter.

Based on this initial concentration of *C. albicans*, there was a 3 Log_10_ microbial reduction in the aPDT groups treated with 0.6% and 1.0% (w/v) gellan gum containing methylene blue (50 μM) and the aqueous group of methylene blue (50 μM). These results indicated that methylene blue incorporated into hydrogels of both concentrations showed a photodynamic action similar to that of the aPDT group with methylene blue in an aqueous solution. In studies along the same lines, a reduction of 3 Log_10_ was also found in a planktonic culture of clinical isolates of *Candida auris*, resistant to fluconazole and caspofungin, after photodynamic therapy mediated by aqueous methylene blue at concentrations of 25, 50, and 100 μM. Likely, in the murine oral candidiasis model, photodithazine-mediated aPDT significantly reduced the colony count by approximately 3 Log_10_.^46^ Despite the similar reduction, it should be noted that the authors used different irradiation parameters from those used in this study, such as a power density of 80 mW/cm^2^ and an irradiation time of 120 s^47^

By demonstrating the efficacy of photodynamic therapy, mediated by the MB carrier system in gellan gum, on planktonic cultures of *C. albicans*, this study sought to advance the study of aPDT for biofilms. The ability to form biofilms is a resistance mechanism of *C. albicans*, as it potentiates the resistance of these microorganisms to various antifungal agents due to the formation of the extracellular matrix, the efflux pump, and the presence of persistent cells.^48^ Thus, biofilms are known to be more complex and resistant forms of *C. albicans* compared to a planktonic culture. In the present study, the application of aPDT to the biofilms of *C. albicans* led to a reduction of 1.5 Log_10_ in the group treated with aqueous MB (50 μM) compared to the untreated control, while the groups containing 0.6% and 1.0% (w/v) MB in gellan gum showed reductions of approximately 1 Log_10_. In a similar study, a reduction of 2.9 Log_10_ of *C. albicans* biofilms was obtained after PDT mediated by aqueous methylene blue (600 μM) at concentrations higher than in our study.^49^ Another study demonstrated that at concentrations of 25 μM to 1250 μM of aqueous methylene blue, aPDT showed strong antimicrobial activity against the growth of *C. albicans*.^50^ However, at higher concentrations of MB of up to 1250 μM, the efficacy of aPDT was reduced, probably due to the increase in the light extinction coefficient of the medium, preventing adequate irradiation of the light source in the reaction space.

*G. mellonella* is an alternative model for animal experimentation and is currently being explored in the development of possible treatments for infection in a burn model.^51,52^ This model is attractive from an experimental point of view because it is cheaper, easier to handle and care for, has a fast life cycle, and does not require committee ethical approval, compared to mammalian models such as mice and rabbits.^53,54^ In a recent study,^55^ our research group used this model as an experiment of burns and infection by a multiresistant strain of *Acinetobacter baumannii* treated by aPDT mediated by the chlorin e–6 photosensitizer compared to aqueous methylene blue. Under similar irradiation parameters to the present study, the authors observed 80% survival of the *G. mellonella* burn population treated by aPDT with aqueous MB compared to the control group without intervention. The results demonstrate the effectiveness of aPDT in controlling infection in a *G. mellonella* burn model as well as improved health index results.

In the present study, the results of the survival curve and the health index of *G. mellonella* showed a superior potential ability to treat burn infection with the GG0.6% hydrogel containing methylene blue at 75 μM concentration when compared with those of the aqueous form, which can be explained by the hydrogel’s greater retention capacity on the burn surface. The difficulty in applying aPDT topically is the low retention of the photosensitizer at the application site. Enabling the delivery system of the photosensitizer in the form of a hydrogel allows for greater contact time and retention on the surface to be treated, resulting in more effective photodynamic therapy when compared to its aqueous form.^56,57^ In clinical practice, a longer retention time of the photosensitizer is required for its better penetration into biofilms.^58^

Since aPDT mediated by methylene blue gellan formulations showed great antifungal activity against *C. albicans* in both in vitro and in vivo models, future studies can be developed to explore its mechanisms of action on fungal cells and to extend its effects for bacterial cells. Reactive oxygen species (ROS) and osmotic disturbances are considered stressors for *C. albicans* cells. In response, the pathogen activates the HOG pathway, which triggers a MAP kinase cascade culminating in the phosphorylation of HOG1. Activated HOG1 promotes the expression of antioxidant enzymes such as catalase and glutathione reductase, which defend the cell against oxidative damage.^59,60^ This signaling pathway enhances the pathogenicity of *C. albicans* by increasing its resistance to oxidative and osmotic stress, promoting phenotypic and morphological adaptations that may interfere with the efficacy of antimicrobial photodynamic therapy.^60,61^ Despite this resistance mechanism, the nonspecific action of reactive oxygen species (ROS) generated through the photoactivation of the photosensitizer, the gellan gum hydrogel shows strong potential for use in aPDT targeting Gram-positive and Gram-negative bacteria, as previously demonstrated in studies employing alternative photosensitizer delivery systems.^62,63^ Considering that the previously described HOG1 pathway is present in all eukaryotic cells, it can be assumed that ROS generated by antimicrobial photodynamic therapy would be more effective in bacterial cells, as these cells lack this environmental adaptation mechanism.^61^

In general, this study presents important preliminary findings; however, some limitations must be acknowledged. First, the methylene blue-loaded gellan gum hydrogel was tested exclusively against *C. albicans* strains. To fully validate the antimicrobial efficacy of this platform for aPDT, further studies are required involving other clinically relevant pathogens, particularly bacterial species commonly associated with skin and mucosal infections. Second, although survival curves in the *G. mellonella* model demonstrated promising outcomes, more in-depth investigations are necessary to elucidate the underlying immune responses and histopathological changes associated with the gellan gum hydrogel with methylene blue-mediated aPDT. These analyses would provide critical insights into the mechanisms of action and safety profile of the treatment. Lastly, despite the success of the burn wound infection model, potential applications of this therapy in other clinically relevant scenarios remain to be explored. For instance, future studies should evaluate its effectiveness in mammalian models of oral candidiasis in which environmental factors such as the microbiota and immune systems of the host must be considered.

As perspectives, the well-succeeded results with the use of gellan gum instigate studies for elucidating the mechanisms of action involved in the microbial inactivation, such as analysis of reactive oxygen species and damage to cellular targets. Future research is also incited to investigate the potential of this hydrogel system to deliver other photosensitizers and broaden its application across a wide range of infections caused by drug-resistant strains. Further, in vivo and pharmacokinetic studies are necessary to establish the safety, efficacy, and practical benefits of these formulations in real-time clinical scenarios.

## 5 Conclusion

In conclusion, this study demonstrates the potential of gellan gum as an effective drug carrier for the photosensitizer methylene blue targeted to antifungal photodynamic therapy. The gellan gum formulations were able to incorporate MB, maintain its optical properties, and provide a controlled release. The antifungal activity of the formulations in aPDT was confirmed against *C. albicans* in various stages, including planktonic growth, biofilms, and in vivo infection. Promisingly, aPDT with MB gellan formulations showed a higher efficacy to treat wound *Candida* infections than MB aqueous. Then, the final product developed in this study enhances the application of aPDT in body areas where photosensitizer retention is challenging, making MB-gellan formulations viable alternatives for aPDT in several medical and dental areas.
